# Autofluorescence lifetime imaging classifies human B and NK cell activation state

**DOI:** 10.3389/fbioe.2025.1557021

**Published:** 2025-04-04

**Authors:** Rebecca L. Schmitz, Jeremiah M. Riendeau, Kelsey E. Tweed, Peter Rehani, Kayvan Samimi, Dan L. Pham, Isabel Jones, Elizabeth M. Maly, Emmanuel Contreras Guzman, Matthew H. Forsberg, Ankita Shahi, Lucia Hockerman, Jose M. Ayuso, Christian M. Capitini, Alex J. Walsh, Melissa C. Skala

**Affiliations:** ^1^ Morgridge Institute for Research, Madison, WI, United States; ^2^ Department of Biomedical Engineering, University of Wisconsin, Madison, WI, United States; ^3^ Department of Pediatrics, University of Wisconsin School of Medicine and Public Health, Madison, WI, United States; ^4^ Carbone Cancer Center, University of Wisconsin School of Medicine and Public Health, Madison, WI, United States; ^5^ Department of Dermatology, University of Wisconsin, Madison, WI, United States

**Keywords:** NK cells, B cells, autofluorescence, imaging, Immune activation

## Abstract

New non-destructive tools with single-cell resolution are needed to reliably assess B cell and NK cell function for applications including adoptive cell therapy and immune profiling. Optical metabolic imaging (OMI) is a label-free method that measures the autofluorescence intensity and lifetime of the metabolic cofactors NAD(P)H and FAD to quantify metabolism at a single-cell level. Here, we demonstrate that OMI can resolve metabolic changes between primary human quiescent and IL-4/anti-CD40 activated B cells and between quiescent and IL-12/IL-15/IL-18 activated NK cells. We found that stimulated B and NK cells had an increased proportion of free compared to protein-bound NAD(P)H, a reduced redox state, and produced more lactate compared to control cells. The NAD(P)H mean fluorescence lifetime decreased in the stimulated B and NK cells compared to control cells. Random forest models classified B cells and NK cells according to activation state (CD69^+^) based on OMI variables with an accuracy of 93%. Our results show that autofluorescence lifetime imaging can accurately assess B and NK cell activation in a label-free, non-destructive manner.

## Introduction

Originating from lymphoid progenitor cells, lymphocytes are a class of immune cells comprised of T cells, B cells, and natural killer (NK) cells (Omman et al., 2020). As a group, lymphocytes account for 20%–40% of circulating white blood cells and are primarily involved in the adaptive immune response which provides specific targeting and immune memory, allowing for long-term protection (Omman et al., 2020). Like T cells, NK cells show cytotoxicity and immune-modulating activities after activation and are emerging in early phase trials as a viable adoptive cell therapy for cancer (Vivier et al., 2008; Cho et al., 2020), particularly using cytokine induced memory-like NK cells (Romee et al., 2016). In contrast to NK and T cells, B cells are primarily responsible for producing antibodies (Bonilla and Oettgen, 2010; Wennhold et al., 2019), but can also act as antigen-presenting cells that promote T cell effector functions (Hoffman et al., 2016; Bonilla and Oettgen, 2010). Additionally, B cells are comprised of various subsets that can either attenuate or suppress the function of surrounding immune cells (Hung et al., 2018). Together, these functions provide several avenues for leveraging B cells as a platform for cell-based therapies, such as antigen-presenting B cells in cancer immunotherapy (Hung et al., 2018; Wennhold et al., 2019). To assess the potency of these cell therapies, lymphocytes are typically exposed to stimuli including cytokines and antigens, followed by response measurement via functional profiling techniques (Cho et al., 2020; Vendrame et al., 2020; Pasero et al., 2015; van der Leun et al., 2020; Tsioris et al., 2015; Mpande et al., 2021).

Current functional profiling techniques for lymphocytes include flow cytometry, cytokine release assays, single-cell RNA sequencing, and cytometry by time of flight (CyTOF). Flow cytometry provides single-cell resolution, but requires labelling with fluorescent antibodies that can be time consuming, may be disruptive to cells, and complicates further use of cells (Boonyaratanakornkit et al., 2019). Bulk measurements of cytokine release are also popular but do not routinely provide single-cell measurements, and ELISPOT, which provides single-cell cytokine release information also requires cell labeling (Boonyaratanakornkit et al., 2019). Finally, single-cell RNA sequencing and CyTOF provide extensive single-cell information, but destroy the sample (Vendrame et al., 2020; Chen et al., 2019). In this work, we demonstrate autofluorescence intensity and lifetime imaging as a compelling alternative to assess B and NK cell function. Unlike traditional methods, autofluorescence imaging leverages endogenous sources of contrast, providing a real-time, non-destructive observation of cellular activation dynamics (Datta et al., 2020). This approach not only preserves cell viability for subsequent analysis but also provides insights into metabolic heterogeneity between cells, making it a powerful tool for advancing immunological research (Suhling et al., 2005; Lemire et al., 2022; Miskolci et al., 2022; Heaster et al., 2020; Yakimov et al., 2019).

Optical metabolic imaging (OMI) is an autofluorescence imaging technique that measures the fluorescence intensities and lifetimes of the reduced metabolic redox cofactors nicotinamide adenine dinucleotide and its phosphorylated counterpart (denoted as NAD(P)H due to their overlapping spectral properties), and the oxidized metabolic redox cofactor, flavin adenine dinucleotide (FAD) (Skala et al., 2007; Georgakoudi and Quinn, 2012; Huang et al., 2002; Lakowicz et al., 1992). NAD(P)+ and FADH_2_ are not fluorescent. However, since NAD(P)H and FAD redox reactions are coupled, the intensities of NAD(P)H and FAD can be combined to provide information on the redox state within the cell (Alfonso-García et al., 2016; Skala et al., 2007; Chance et al., 1979; Ostrander et al., 2010; Walsh and Skala, 2015). There are many definitions of the optical redox ratio, though here we define the optical redox ratio as the fluorescence intensities of NAD(P)H/(NAD(P)H + FAD). Additionally, the fluorescence lifetimes of NAD(P)H and FAD are distinct in the free and protein-bound conformations, where multi-exponential decay fits recover τ_1_ as the fast decay (free NAD(P)H, bound FAD) and τ_2_ as the long decay (bound NAD(P)H, free FAD) (Lakowicz et al., 1992; Sharick et al., 2018; Schaefer et al., 2019; Nakashima et al., 1980). The relative proportion of each short and long lifetime component are recovered as α_1_ and α_2_, respectively.

OMI is a promising technique to evaluate lymphocyte activation because known metabolic shifts occur with activation in NK, B, and T cells. Unstimulated lymphocytes have low metabolic demands and largely rely on low levels of glycolysis and oxidative phosphorylation to generate ATP (Cong, 2020; Gardiner and Finlay, 2017; Ripperger and Bhattacharya, 2021; Donnelly et al., 2014). To fuel rapid proliferation, cytokine production, and other effector functions, activated lymphocytes increase energy production through aerobic glycolysis and oxidative phosphorylation (Gardiner and Finlay, 2017; Chapman and Chi, 2022; O'brien et al., 2019). Overnight stimulation with activating cytokines (including IL-2, IL-12, and IL-15) increases rates of glycolysis and oxidative phosphorylation in NK cells (Keating et al., 2016; Donnelly et al., 2014). Similar increases in glycolysis and oxidative phosphorylation also occur with activation in B and T cells (Ripperger and Bhattacharya, 2021; Chapman and Chi, 2022).

Prior studies have characterized changes in OMI variables with T cell function, particularly the correlation between increased glycolytic demand and elevated levels of free intracellular NAD(P)H (α_1_) due to T cell activation (Hu et al., 2020; Walsh et al., 2021; Paillon et al., 2024). However, the ability of OMI to discriminate quiescence and activation in B and NK cells remains less explored. Here, we demonstrate that OMI provides a label-free and single-cell method to evaluate metabolic changes with activation of B and NK cells. This metabolic imaging approach could be used to monitor lymphocyte function in a label-free and single-cell manner for applications where non-invasive single-cell monitoring is critical (e.g., adoptive cell therapy manufacturing, monitoring changes over time within intact systems).

## Results

### OMI resolves metabolic differences between quiescent and activated primary human B cells

**FIGURE 1:**
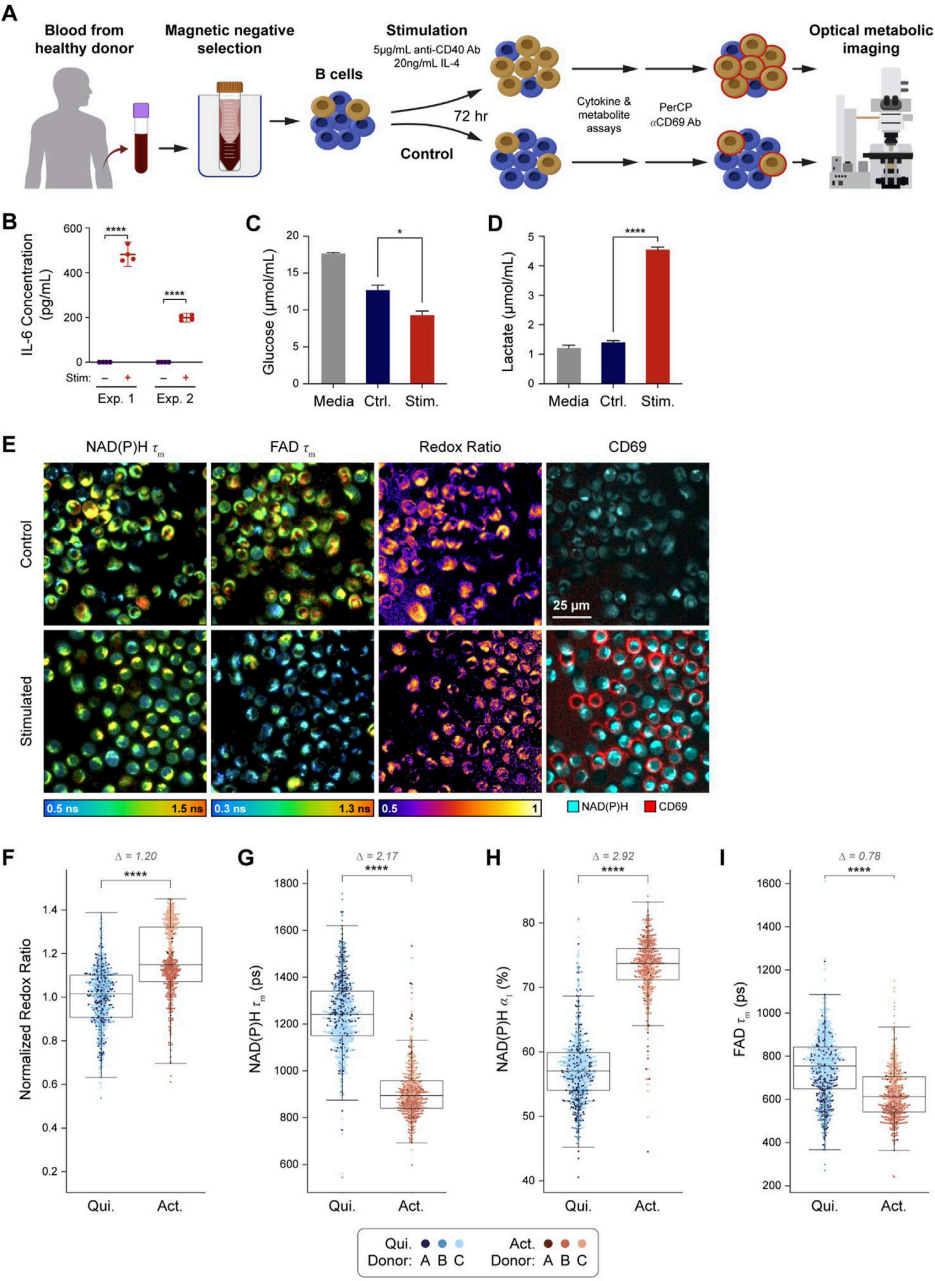
OMI is sensitive to metabolic changes between primary human B cells that are quiescent vs activated with IL-4 and anti-CD40. **(A)** B cells were isolated from human peripheral blood of three different donors **(A-C)** and stimulated for 72 h with 5 μg/mL anti-CD40 and 20 ng/mL IL-4 or cultured in just Advanced RPMI-1640 + 5%FBS media (control). Media samples were collected after 72 h of culture. **(B)** IL-6 concentration was measured from media samples of two different donors cultured in stimulated or control conditions for 72 h. **(C)** Glucose in the media of stimulated B cells was significantly decreased compared to the control cell media after 72 h of culture. **(D)** Lactate levels in stimulated B cell media were significantly higher than lactate levels in the control B cell media. **(E)** Representative images of NAD(P)H τ_m_, FAD τ_m_, redox ratio, and anti-CD69 staining in the control and stimulated conditions. **(F)** Redox ratio normalized to the mean of the quiescent group for each donor significantly increased in activated B cells (CD69^+^ in stimulated media) compared to quiescent B cells (CD69^−^in control media). **(G, H)** NAD(P)H τ_m_ significantly decreased and NAD(P)H α_1_ significantly increased in the activated B cells compared to the quiescent B cells. **(I)** A significant decrease in FAD τ_m_ was observed in the activated B cells compared to quiescent B cells. In **F-I**, data are displayed as box-and-whisker plots, representing the median and interquartile range (IQR), with whiskers at 1.5*IQR Glass’s Delta measure of effect size given for Δ. Plots are overlaid with data points; each point represents 1 cell, color coded by donor **(A–C)**. n = 1,210 cells (461 activated B cells, 749 quiescent B cells). *P<0.05, **** P < 0.0001, two-tailed unpaired T-test.

A graphical overview of the experimental design is provided (Figure 1A). Isolated primary human B cells from three donors were stimulated using anti-CD40 antibody and IL-4 to mimic T cell mediated activation (Wennhold et al., 2019; Janeway et al., 2001; Akkaya et al., 2020). After 72 h of *in vitro* activation, media was collected for cytokine, glucose, and lactate assays, then cells were stained with anti-CD69 antibody to identify activated and quiescent cells in each condition for subsequent OMI. To confirm that our protocol successfully stimulated the B cells, the concentration of IL-6 in the media was measured at 72 h and found to be significantly higher in the stimulated compared to the control condition (Figure 1B). Additionally, analysis of glucose and lactate levels in the spent media at 72 h show decreased glucose and increased lactate in the media of stimulated B cells compared to control, suggesting increased glucose consumption and glycolytic activity in stimulated cells (Figures 1C, D). These media measurements are consistent with known metabolic changes upon B cell activation (Ripperger and Bhattacharya, 2021; Chapman and Chi, 2022). Representative images from OMI are shown in Figure 1E, which include NAD(P)H mean fluorescence lifetime (τ_m_), FAD τ_m_, optical redox ratio, and pseudocolored CD69 fluorescence. Qualitatively, most B cells in the stimulated condition stained positive for CD69 while few stained positive in the control condition.

For all our experiments, quiescent cells were defined as CD69^−^cells in the control condition, while activated cells were defined as CD69^+^ cells in the stimulated condition. The optical redox ratio (ORR = I_NAD(P)H_/[I_NAD(P)H_ + I_FAD_]) was elevated in activated B cells compared to quiescent B cells (Figure 1F). Additionally, NAD(P)H τ_m_ and FAD τ_m_ decreased while NAD(P)H α_1_ (the fraction of free NAD(P)H) increased in activated B cells compared to the quiescent B cells (Figures 1G–I). These changes were consistent with the higher glycolytic activity previously reported for activated B cells (Ripperger and Bhattacharya, 2021; Lawlor et al., 2021). These OMI changes are also consistent with our glucose and lactate measurements (Figures 1C, D).

When comparing CD69^+^ and CD69^−^cells within the control condition, there were no significant differences in any OMI variables (Supplementary Figure 1). However, in the stimulated condition, CD69^+^ cells were significantly different compared to CD69^−^cells for all OMI variables except for the optical redox ratio (Supplementary Figure 1).

### Single cell clustering and machine learning models based on OMI separate B cells by activation state

**FIGURE 2:**
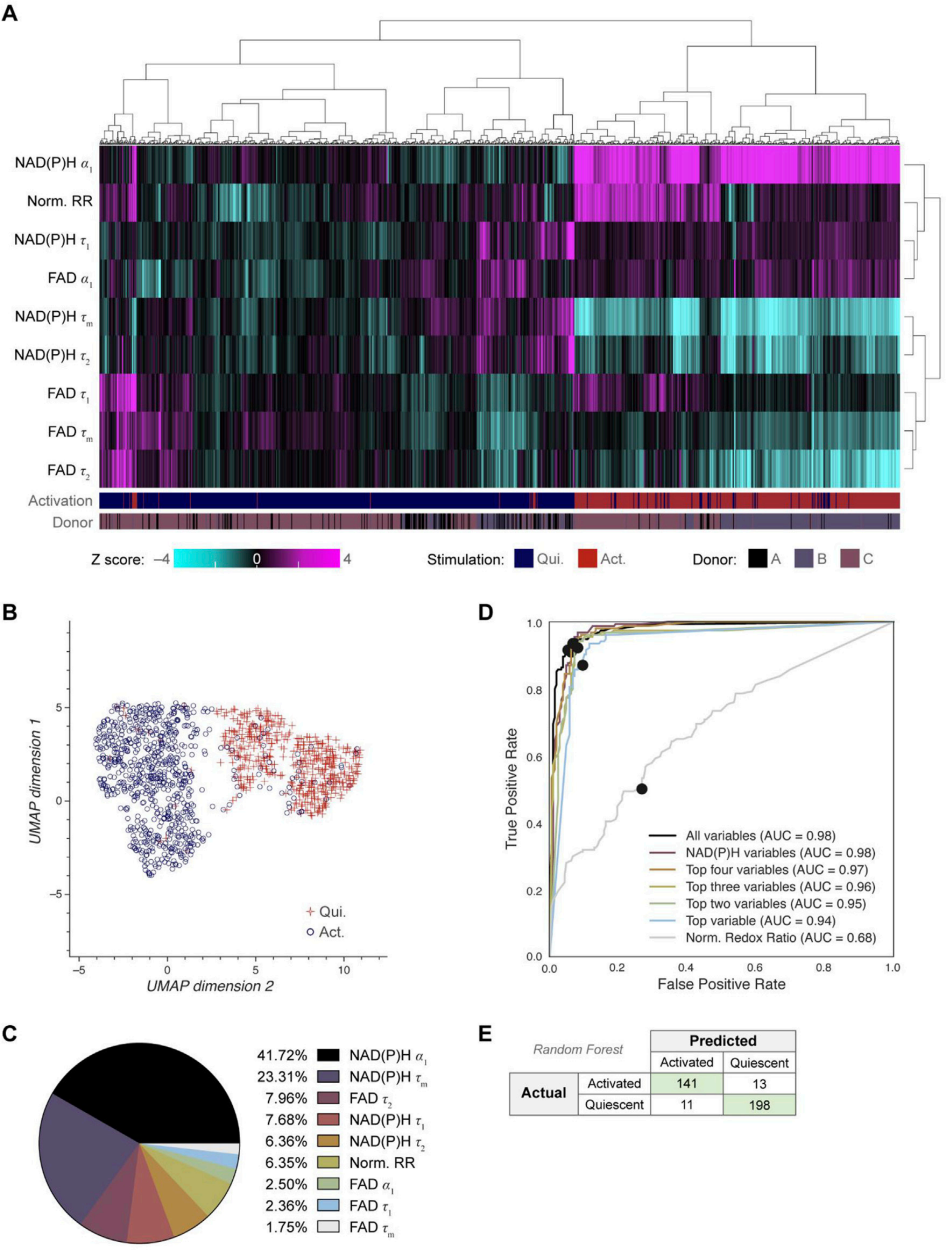
OMI characterizes single cell heterogeneity and accurately classifies activated from quiescent B cells. **(A)** Heatmap of single-cell OMI variables across all B cell experiments. Hierarchical cell clustering was calculated based on the z-scores (the difference between cell mean and population mean divided by the population standard deviation) for nine OMI variables (NAD(P)H τ_m_, τ_1_, τ_2_, α_1_; FAD τ_m_, τ_1_, τ_2_, α_1_; and quiescent-normalized optical redox ratio). Activated B cells cluster separately from quiescent B cells regardless of donor. **(B)** UMAP of nine OMI variables visualizes separation between clusters of activated and quiescent B cells. **(C)** Pie chart of the relative weight of the nine OMI variables included in the “all variables” random forest classifier. **(D)** Receiver operating characteristic (ROC) curve of random forest classifiers trained on different combinations of OMI variables to classify quiescent and activated B cells, with operating points indicated. “Top variables” classifiers refer to the largest weighted variables in the “all variable” classifier, found in **(C)**. The classifiers using all the variables or only the NAD(P)H variables (NAD(P)H τ_m_, τ_1_, τ_2_, α_1_) performed best (AUC 0.98), followed by the classifier that used the top four OMI variables (AUC 0.97), three of which are NADH(P)H lifetime variables. **(E)** Confusion matrix of the nine OMI variables random forest classifier shows performance for classification of activated and quiescent B cells with an accuracy score of 0.934. n = 1,210 cells (461 activated B cells, 749 quiescent B cells) with a 70/30 split for training and test sets.

Next, we investigated whether OMI could visualize single cell heterogeneity in B cells and whether machine learning models based on OMI could classify B cell activation state. Unsupervised clustering using the 9 OMI variables of activated and quiescent cells revealed that the activated cells cluster separately from the quiescent cells across all three donors (Figure 2A). Uniform manifold approximation and projection (UMAP) was used to visualize the clustering of single B cells based on the same OMI variables, which similarly revealed distinct clusters of activated and quiescent cells (Figure 2B). Additionally, a UMAP colored by activation (activated, quiescent) and donor are provided (Supplementary Figure 2A).

Next, a random forest classifier based on OMI variables for each B cell was trained on 70% of the cells and tested on the remaining 30% of cells to identify activated and quiescent B cells. The OMI variables with the greatest weight in the classification of activated and quiescent B cells were NAD(P)H α_1_ (41.72%), NAD(P)H τ_m_ (23.31%), free FAD fluorescence lifetime (τ_2_) (7.96%), and free NAD(P)H fluorescence lifetime (τ_1_) (7.68%) (Figure 2C). Random forest classifiers performed best when given all variables or only NAD(P)H variables (AUC = 0.98), revealing that NAD(P)H is most informative for identifying B cell activation in these conditions (Figure 2D). The resulting confusion matrix has an accuracy of 0.934 (Figure 2E). Alternatively, a Fourier transform can be used to represent the fluorescence decay in frequency space, which is known as a phasor representation (Digman et al., 2008). The lifetime decay variables in the time domain and the phasor representation in the frequency domain both represent the same underlying fluorescence decay, so a classifier based on phasor variables should perform similarly to that based on lifetime decay variables (Digman et al., 2008). Classification based on the NAD(P)H and FAD phasors at both the laser repetition frequency (80 MHz) and its second harmonic (160 MHz) predicted B cell activation with 0.939 accuracy (Supplementary Figures 2B-D).

### OMI resolves metabolic differences between quiescent and activated primary human NK cells

**FIGURE 3:**
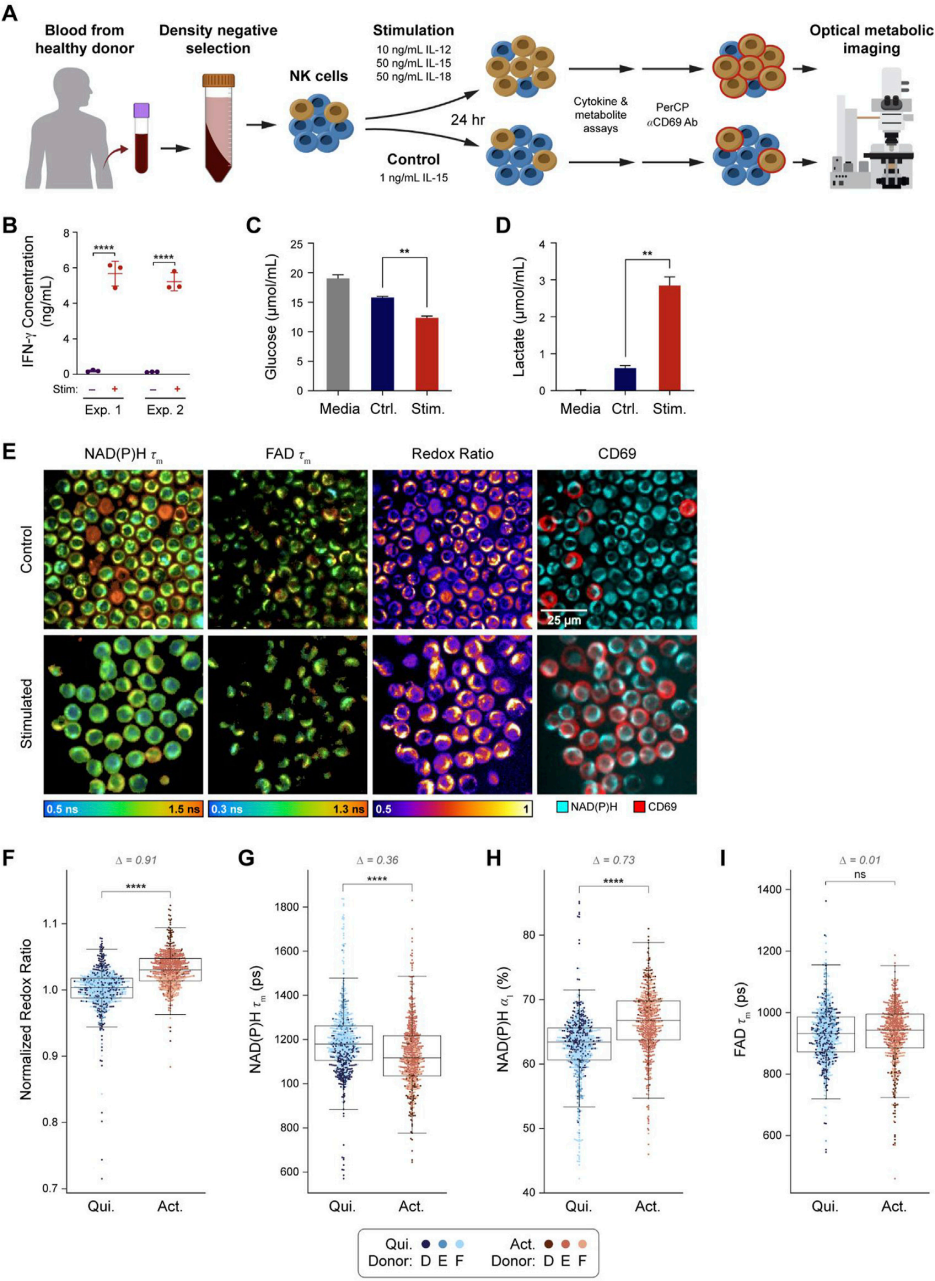
OMI is sensitive to metabolic changes between primary human NK cells that are quiescent vs activated with IL-12, IL-15, and IL-18. **(A)** NK cells were isolated from human peripheral blood of three different donors **(D-F)** and stimulated for 24 h with 10 ng/mL IL-12, 50 ng/mL IL-15, and 50 ng/mL IL-18 or cultured in just TheraPeak X-VIVO-10 medium+10% human serum AB+ 1 ng/mL IL-15 (control). **(B)** IFN-γ concentration was measured from media samples of two different donors cultured in stimulated or control conditions for 24 h. **(C)** Glucose in the media of stimulated NK cells was significantly decreased compared to the control cell media after 24 h of culture. **(D)** Lactate levels in stimulated B cell media were significantly higher than lactate levels in the control cell media. **(E)** Representative images of NAD(P)H τ_m_, FAD τ_m_, redox ratio, and anti-CD69 staining in the control and stimulated conditions. **(F)** Redox ratio normalized to the mean of the quiescent group for each donor significantly increased in activated (CD69^+^ in stimulated media) NK cells compared to quiescent (CD69^−^in control media) NK cells. **(G, H)** NAD(P)H τ_m_ significantly decreased and NAD(P)H α_1_ significantly increased in the activated NK cells compared to the quiescent NK cells. **(I)** No change in FAD τ_m_ was observed in the activated NK cells compared to quiescent NK cells. In **(F–I)**, data are displayed as box-and-whisker plots, representing the median and interquartile range (IQR), with whiskers at 1.5*IQR. Glass’s Delta measure of effect size given for Δ. Plots are overlaid with data points; each point represents 1 cell, color coded by donor **(D–F)**. n = 1,221 cells (554 activated NK cells, 667 control NK cells). **P<0.01, **** P < 0.0001, two-tailed unpaired T-test. ns = not significant.

A graphical overview of the NK experiment is provided (Figure 3A). Isolated primary human NK cells from three donors were stimulated *in vitro* for 24 h using IL-12, IL-15, and IL-18 as previously described for inducing memory-like NK cells (Romee et al., 2016; Cooper et al., 2009; Ni et al., 2012; Gang et al., 2020). After 24 h of *in vitro* activation, media was collected for cytokine, glucose, and lactate assays, then cells were stained with anti-CD69 antibody to identify activated and quiescent cells for OMI analysis. To confirm NK cell stimulation, the concentration of IFN-γ in the media was measured at 24 h and found to be significantly increased in the stimulated NK cell media when compared to control NK cell media (Figure 3B). Similarly, analysis of glucose and lactate levels at 24 h show decreased glucose and increased lactate in the media of stimulated compared to control NK cells (Figures 3C, D), confirming known metabolic changes with NK cell activation (O’Brien and Finlay, 2019; Donnelly et al., 2014; Keating et al., 2016). Representative images of NAD(P)H τ_m_, FAD τ_m_, optical redox ratio, and pseudocolored CD69 expression are presented (Figure 3E). Qualitatively, most NK cells in the stimulated condition stained positive for CD69 while few stained positive in the control condition.

OMI revealed several changes with activation in NK cells. The optical redox ratio significantly increased in activated NK cells compared to quiescent NK cells (Figure 3F). NAD(P)H τ_m_ decreased, and NAD(P)H α_1_ increased in activated NK cells compared to quiescent NK cells (Figures 3G–I). Within stimulated or control media, OMI variables did not change with CD69 status except for one variable; in the control condition, NAD(P)H τ_1_ was slightly lower in CD69^+^ NK cells *versus* the CD69^−^ NK cells (Supplementary Figure 3).

### Single cell clustering and machine learning models based on OMI separate NK cells by activation state

**FIGURE 4:**
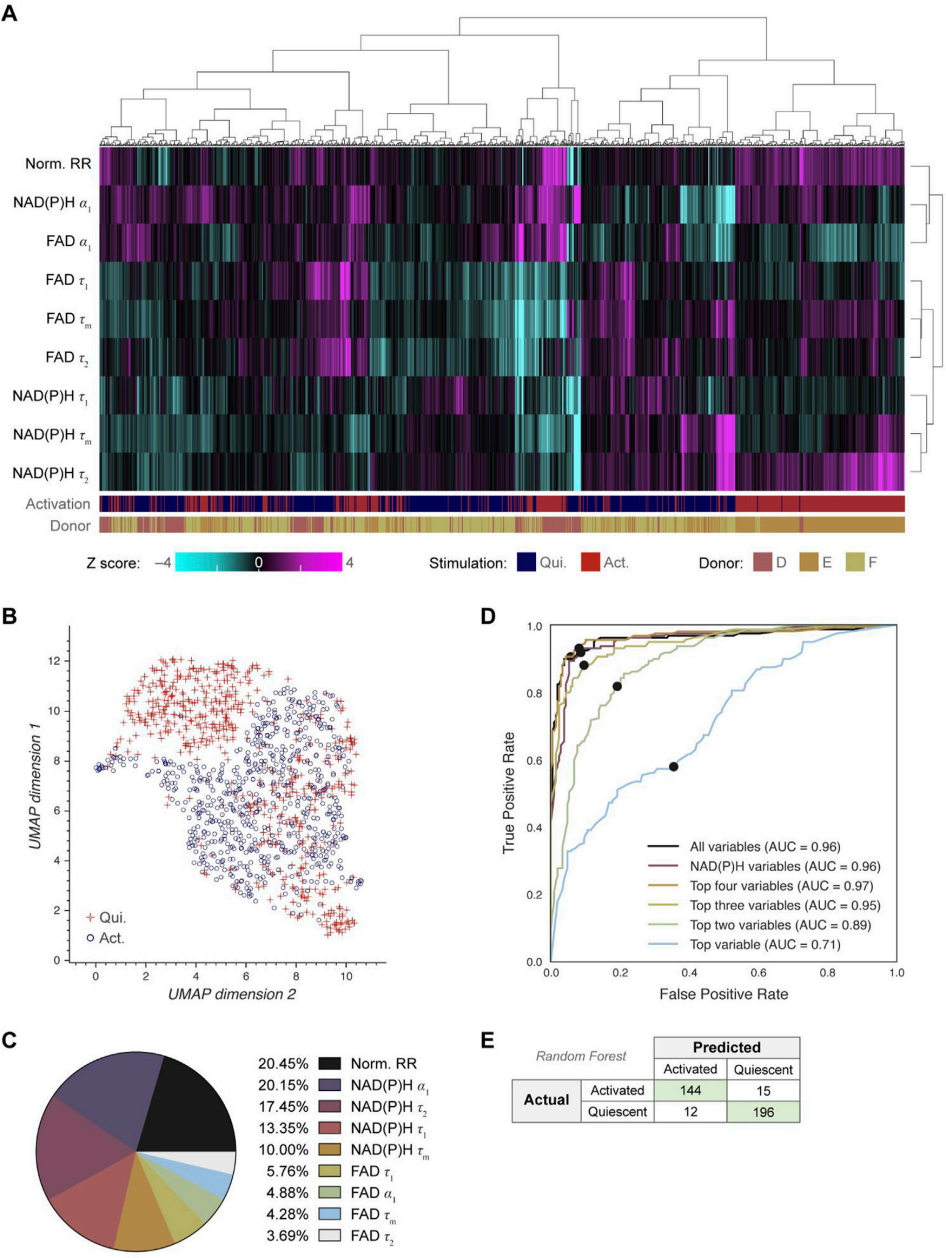
OMI characterizes single cell heterogeneity and accurately classifies activated from quiescent NK cells. **(A)** Heatmap of single-cell OMI variables across all NK cell experiments. Hierarchical cell clustering was calculated based on the z-scores (the difference between cell mean and population mean divided by the population standard deviation) of nine OMI variables (NAD(P)H τ_m_, τ_1_, τ_2_, α_1_; FAD τ_m_, τ_1_, τ_2_, α_1_; and quiescent-normalized optical redox ratio). **(B)** UMAP of nine OMI variables displays clustering of activated and quiescent NK cells. **(C)** Pie chart of the relative weight of the nine OMI variables included in the “all variables” random forest classifier. **(D)** Receiver operating characteristic (ROC) curve of random forest classifiers trained on different combinations of OMI variables to classify quiescent and activated NK cells, with operating points indicated. “Top variables” classifiers refer to the largest weighted variables in the “all variable” classifier, found in **(C)**. The classifier using the top four OMI variables performed the best (AUC 0.97), followed by the classifier that used all nine OMI variables (AUC 0.96) and the classifier that used only NAD(P)H lifetime variables (NAD(P)H τ_m_, τ_1_, τ_2_, α_1_) (AUC 0.96). **(E)** Confusion matrix of the nine OMI variables random forest classifier shows performance for classification of activated and quiescent NK cells with an accuracy score of 0.926. n = 1,221 cells (554 activated NK cells, 667 quiescent NK cells) with a 70/30 split for training and test sets.

Next, we investigated whether OMI could visualize single cell heterogeneity in NK cells and whether machine learning models based on OMI can classify NK cell activation state. Unsupervised clustering of the 9 OMI variables from quiescent and activated cells revealed that NK cells were somewhat heterogeneous, resulting in the emergence of a dominant cluster with several smaller clusters of activated and quiescent cells (Figure 4A; Supplementary Figure 4A). UMAP was used to visualize the clustering of single NK cells based on the same OMI variables, which demonstrated a cluster of activated NK cells separate from a mixed cluster of activated and quiescent NK cells (Figure 4B).

A random forest classifier based on single-cell OMI variables was trained and tested on 70% and 30%, respectively, of the NK cells to identify activated or quiescent cell states. The highest weighted OMI variables were the optical redox ratio (20.45%), NAD(P)H α_1_ (20.15%), protein-bound NAD(P)H fluorescence lifetime (τ_2_) (17.45%), and free NAD(P)H fluorescence lifetime (τ_1_) (13.35%) (Figure 4C). Random forest classifiers performed best (AUC = 0.97) when given the top four variables, which include the optical redox ratio and three NAD(P)H lifetime variables (NAD(P)H τ_1_, τ_2_, α_1_), revealing that NAD(P)H is most informative for identifying NK cell activation in these conditions (Figure 4D). The resulting confusion matrix has an accuracy of 0.926 (Figure 2E). Classification based on the NAD(P)H and FAD phasors at both the laser repetition frequency (80 MHz) and its second harmonic (160 MHz) predicted NK cell activation with a similar accuracy of 0.892 (Supplementary Figures 4B-D).

### Single cell heterogeneity of activated and quiescent B and NK cells

Single cell heterogeneity within activated and quiescent B and NK cells was summarized with the coefficient of variation (Supplementary Figure 5). NAD(P)H τ_1_ was heterogeneous across B cells in the quiescent state but became more homogeneous with B cell activation. Additionally, FAD τ_m_ was heterogeneous across both quiescent and activated B cells, which is driven by heterogeneous FAD τ_1_ in these cells (Supplementary Figure 5). This heterogeneity in FAD τ_m_ and τ_1_ within quiescent and activated B cells may explain their poor predictive power in classifying B cell activation (Figure 2C). Similarly, FAD τ_1_ within quiescent and activated NK cells demonstrated relatively high heterogeneity, indicating that protein-bound FAD drives the observed heterogeneity in autofluorescence variables across B and NK cells.

## Discussion

Several areas of research and clinical care rely on lymphocyte functional assessments and would benefit from a non-destructive, single-cell, touch-free technology to assess lymphocyte activation state, which could reduce the cost and time for analysis. In this report, we have demonstrated that OMI is sensitive to metabolic changes that occur with activation in primary human B cells and NK cells. Additionally, machine learning models trained on single-cell OMI variables reliably classify quiescent B cells from IL-4/anti-CD40 activated B cells and quiescent NK cells from IL-12/IL-15/IL-18 activated memory-like NK cells.

Importantly, we observed consistent changes in OMI variables with activation in B cells and NK cells, including increased optical redox ratio, decreased NAD(P)H τ_m_, and increased NAD(P)H α_1_, which is consistent with prior work on primary human T cells (Hu et al., 2020; Walsh et al., 2021; Paillon et al., 2024). These observations suggest a conserved metabolic shift when lymphocytes become activated, consistent with prior studies demonstrating that T, B, and NK cells upregulate oxidative phosphorylation and aerobic glycolysis when activated to fuel effector functions, rapid growth, and proliferation (Chapman and Chi, 2022; Donnelly et al., 2014; Keating et al., 2016; Assmann et al., 2017; Poznanski et al., 2018). Previous studies have demonstrated that OMI is sensitive to metabolic pathway preferences in the cell (Sharick et al., 2018; Walsh et al., 2013). In this report, measurements of glucose and lactate levels in media from control and stimulated B and NK cells revealed that the glucose concentration significantly decreased whereas the lactate concentration significantly increased with activation. This observation is consistent with prior studies that observed an upregulation of aerobic glycolysis with B and NK cell activation, which underlies changes in OMI variables (Lawlor et al., 2021).

The single-cell resolution of OMI makes it a powerful tool for characterizing differences within a population. Here, we characterized changes within control and stimulated B cell and NK cell populations. We observed that there was a mixture of CD69^+^ and CD69^−^cells within both control and stimulated groups, and fluorescence lifetime variables were consistently different between CD69^−^and CD69^+^ B cells in the stimulated condition (Supplementary Figure 1), while only NAD(P)H τ_1_ differed between CD69^−^and CD69^+^ NK cells in the control group only (Supplementary Figure 3). These observations indicate that OMI provides similar, but not identical information to CD69 status, which will be explored in future work. In this work, we chose to focus the single-cell analysis on cells that we could confirm to be quiescent (i.e., CD69^−^cells in the control condition) and cells that we could confirm to be activated (i.e., CD69^+^ cells in the stimulated condition) to better characterize the ability of OMI to assess these cells without complications that could arise from the absence of the transiently expressed CD69 marker.

Consistent with our time-domain findings, phasor analysis revealed expected changes in B cell localization within the phase diagram, primarily along the line connecting the two components of NAD(P)H fluorescence decay (Supplementary Figure 2B). This observation aligns with our ROC classification results, where NAD(P)H α_1_ alone accurately predicted B cell activation state (ROC AUC = 0.94). This high predictive performance is expected, given the large effect size for NAD(P)H α_1_ upon activation (Glass’s delta = 2.92). In contrast, NK cell phasor analysis showed differences that were not aligned with the primary axis but rather perpendicular to it (Supplementary Figure 4B). Since the effect size for NAD(P)H α_1_ in NK cell activation was smaller (Glass’s delta = 0.73), we anticipated that additional features would be required for accurate classification. This expectation is supported by our ROC classifier results, where single-variable classification performed poorly (ROC AUC = 0.71).

Although a complete set of NAD(P)H and FAD intensities and lifetimes were collected in this study, all 9 OMI variables may not be necessary for accurate classification. NAD(P)H lifetime variables alone accurately classified activation within B cells (AUC 0.98) and activation within NK cells (AUC 0.96) (Figure 2D; Figure 3D). This indicates that simplified hardware with only NAD(P)H excitation and emission capabilities would perform as accurately as a two-color NAD(P)H and FAD imaging system, which is an important consideration in the design of simplified hardware for use in clinical labs. Additionally, we have recently demonstrated that NAD(P)H lifetime measurements can be reliably performed in a flow geometry, which improves throughput and automation for real-time single-cell analysis (Samimi et al., 2024).

Unlike flow cytometry, OMI is relatively new and does not provide traditional phenotyping based on surface markers. Therefore, OMI is not appropriate for studies where high depth molecular information is needed. High-content single-cell analysis is better performed with flow cytometry, CyTOF, and/or single-cell RNA sequencing. However, OMI is advantageous when single-cell metabolic information is needed from living cells. OMI is also advantageous when touch-free, non-invasive, rapid, and single cell measurements are beneficial, such as continuous monitoring within unperturbed systems (cell culture, 3D culture, *in vivo*), cell therapy production where good manufacturing practice (GMP) must be maintained to generate cells for patient use, and when rapid reactivity tests are needed (*e.g.*, immune profiling).

Overall, these studies demonstrate that OMI can effectively classify B and NK cell activation with single cell resolution in a touch-free manner. This label-free single-cell imaging and classification method allows non-invasive, real-time monitoring of B and NK cell metabolism, which could be used in several settings including rapid assessment of immune responses to stimuli, or cell manufacturing for therapeutic purposes.

## Materials and methods

### Isolation of primary human lymphocytes

Primary human lymphocytes were isolated from peripheral blood of healthy adult donors under approval by the UW-Madison Institutional Review Board. After obtaining informed consent from the donors, 10–50 mL whole blood was drawn using a sterile syringe with heparin. Peripheral blood mononuclear cells were first isolated by diluting peripheral blood with an equal volume of DPBS +2% FBS, then centrifuging at 1,200 × g for 10 min in SepMate tubes containing a layer of Lymphoprep (STEMCELL Technologies). The isolated PBMCs were then washed with DPBS +2% FBS and further processed for specific lymphocyte cell type enrichment. B cells or NK cells were then isolated from PBMCs using negative isolation kits.

For the B or NK cell isolation (EasySep, STEMCELL Technologies), the PBMCs were washed with DPBS +2% FBS and centrifuged at 100 *g* for 10 min. The resulting pellet was resuspended to a concentration of 50 million cells/mL in EasySep Buffer (STEMCELL Technologies). 50 μL/mL isolation cocktail was added to the sample, according to the EasySep protocol and allowed to incubate for 5 min. 50 μL/mL RapidSpheres solution was then added and the mixture was topped to 2.5 mL with DPBS +2% FBS and transferred to a magnet for 3 min. The enriched B or NK cells were poured into a new tube and the sample was again placed into a magnet for 1 min. The enriched cell population was then washed with the respective culture medium and transferred to a cell culture flask or well plate for culture.

### Cell culture and activation

NK cells were cultured in TheraPeak X-VIVO-10 medium (Lonza) supplemented with 10% human serum AB (Sigma Aldrich) and 1 ng/mL IL-15 (Biolegend). B cells were cultured in RPMI containing 5% fetal bovine serum and 1% penicillin-streptomycin. Following isolation, each cell population was divided into two groups: a control population cultured in normal medium, and an activated population cultured in control medium supplemented with additional components. NK cell activating medium was supplemented with 10 ng/mL IL-12 (Invivogen), 50 ng/mL IL-15, and 50 ng/mL IL-18 (Biolegend) (Ni et al., 2012; Cooper et al., 2009). B cell activating medium was supplemented with 5 μg/mL anti-CD40 antibody (R&D systems) and 20 ng/mL IL-4 (R&D Systems) (Wennhold et al., 2019; Van Belle et al., 2016).

The cells were cultured separately in activating or control medium for a number of hours depending on the cell type; B cells were stimulated for 72 h, and NK cells for 24 h (Ni et al., 2012; Cooper et al., 2009; Bonilla and Oettgen, 2010; Van Belle et al., 2016). Cells were seeded at a density of one million cells/mL medium. At the end of the stimulation period, a sample of spent media from each group was taken for cytokine and metabolite analysis. A summary of the isolation and activation conditions used is provided in Table 1.

**TABLE 1:** Isolation and activation conditions for each lymphocyte subtype.

Cell type	Touch-free isolation method	Control medium	Activation medium	Activation time
B cell	EasySep Human Naïve B Cell Isolation Kit (StemCell Technologies)	RPMI +5% FBS +1% penicillin/streptomycin	Control medium +5 μg/mL anti-CD40 antibody + 20 ng/mL IL-4	72 h
NK Cell	EasySep Human NK Cell Isolation Kit (StemCell Technologies)	TheraPeak X-VIVO-10 medium (Lonza) + 10% human serum AB+ 1 ng/mL IL-15	Control medium + 10 ng/mL IL-12 + 50 ng/mL IL-15 + 50 ng/mL IL-18	24 h

### Staining with PerCP conjugated anti-CD69 antibody

At the end of the stimulation period, cells were stained with a PerCP-conjugated anti-CD69 antibody (Biolegend) to distinguish activated from quiescent cells within each condition (Keating et al., 2014; Van Belle et al., 2016). The cells were centrifuged at 300 *g* for 8 min, then resuspended to a concentration of 10 million cells/mL medium. 5μL (200 μg/mL) PerCP-conjugated anti-CD69 antibody per million cells was added to the sample. The cells were then incubated for 30 min at room temperature. Following incubation, the cells were washed twice with media and centrifuged at 300 *g* for 8 min to remove excess antibody from the sample.

### Fluorescence lifetime imaging of B and NK cells

For imaging, cells were plated 1 h before imaging on poly-D-lysine coated glass-bottomed dishes (MatTek) at a seeding density of 200,000 cells in 50 μL media. The cells were imaged with a custom-built multiphoton fluorescence microscope (Ultima, Bruker) using a 100x (NA = 1.45) oil immersion objective and time-correlated single photon counting electronics (SPC-150, Becker and Hickl GbH, Berlin, Germany). The femtosecond-pulsed laser (Insight DS+, Spectra-Physics Inc., Santa Clara, CA, United States) with 80 MHz pulse repetition rate was tuned to 750 nm for NAD(P)H excitation, 890 nm for FAD excitation, and 980 nm or 1,040 nm excitation for PerCP. Fluorescence emission was detected using a H7422PA-40 GaAsP photomultiplier tube (Hamamatsu Corporation, Bridgewater, NJ, United States) and isolated using a 440/80 nm bandpass filter for NAD(P)H, 550/50 nm (NK cells) or 550/100 nm (B cells) bandpass filter for FAD, and 690/50 nm bandpass filter for PerCP. Average power on the sample was kept below 10 mW at 750 nm and below 20 mW at 890 nm to avoid photodamage. The laser power was maintained at a consistent value within each experiment.

270 μm × 270 μm fluorescence lifetime images (256 × 256 pixels) were collected consecutively for NAD(P)H and FAD in the same field of view, with a pixel dwell time of 4.8 μs and an integration time of 60s. An instrument response function was collected during imaging from the second harmonic generation of a urea crystal, and photon count rates were maintained around 1 × 10^5^ photons per second. A matched intensity image of PerCP fluorescence was collected for the same field of view. Images were collected from three to six fields of view for each sample.

### Image analysis

Fluorescence lifetimes were extracted through analysis of the fluorescence decay at each pixel in SPCImage software (Becker and Hickl). To provide more robust calculations of the fluorescence lifetimes, a threshold was used to exclude background pixels with a low intensity, and images were binned up to a bin factor of three to reach a peak of at least 100 photons in the decay. Both NAD(P)H and FAD can exist in a quenched and an unquenched configuration with distinct lifetimes. To extract these lifetimes, fluorescence decays were fit to a two-component exponential decay function that was re-convolved with the instrument response function (Equation 1):

$$I\left(t\right)=\alpha_{1}e^{‐\frac{t}{\tau_{1}}}+\alpha_{2}e^{‐\frac{t}{\tau_{2}}}+C$$ (1)

Where I(t) is the light intensity at time t following the laser pulse, τ_1_ and τ_2_ are the short (quenched) and long (unquenched) lifetimes of the fluorophore, and α_1_ and α_2_ are the fractional proportion of each component. *C* is included to account for background light. For NAD(P)H, the short lifetime (τ_1_) corresponds to free NAD(P)H and the long lifetime (τ_2_) corresponds to protein-bound NAD(P)H (Lakowicz et al., 1992). The opposite is true of FAD: the short and long lifetime correspond to bound FAD and unbound FAD, respectively (Nakashima et al., 1980). A mean lifetime (Equation 2) at each pixel was also computed as the amplitude-weighted average of the short and long lifetimes:

$$\tau_{m}=\left(\alpha_{1}\;\tau_{1}+\alpha_{2}\;\tau_{2}\right)/\left(\alpha_{1}+\alpha_{2}\right)$$ (2)

Following extraction of the fluorescence lifetimes, images were segmented to create single-cell masks using NAD(P)H intensity images. Segmentation was carried out in CellProfiler, resulting in masks of cells, cell nuclei, and cell cytoplasm. PerCP-CD69 fluorescence images were manually segmented by a trained observer. The observer was blinded to whether PerCP-CD69 images came from the activated or control condition. The resulting masks were used to identify activated and quiescent cells in each condition based on 75% overlap between PerCP-CD69 masks and cell masks.

Mean values of fluorescent lifetime components for each cell were calculated in R. The values of NAD(P)H τ_m_, NAD(P)H τ_1_, NAD(P)H τ_2_, NAD(P)H α_1_, FAD τ_m_, FAD τ_1_, FAD τ_2_, and FAD α_1_ were calculated for each cell by averaging across all pixels in the cell cytoplasm. Cells with low photon counts (<5,000 photons), small masks that are unlikely cells (<250 pixels or 75 μm^2^ whole cell area), and pixels with poor goodness-of-fit (χ^2^ > 1.3) were not included in this analysis. In the results, α_1_ and α_2_ refer to the normalized fractions α_1_/(α_1_+ α_2_), and α_2_/(α_1_+ α_2_), respectively, and are presented as percentage values. An additional variable, the optical redox ratio, was computed for each cell, defined here as the NAD(P)H intensity divided by the sum of the NAD(P)H and FAD intensities. This definition of the redox ratio is bound between 0 and 1. To account for variations in intensity from day-to-day equipment and setting changes, the redox ratio of each cell was normalized to the mean redox ratio of the control group for each day.

### Phasor analysis of lifetime images

Phasor representation (Digman et al., 2008) is a fit-free and fast way to reduce a decay histogram (at either pixel or cell level) to a single point on the two-dimensional phasor plot that provides contrast between different fluorescence lifetimes. Mathematically, phasors are the complex coefficients of the Fourier series expansion of the fluorescence decay at harmonics of the excitation laser repetition frequency. The real and imaginary parts of each coefficient are denoted with G (Equation 3) and S (Equation 4), respectively. Usually, the base harmonic carries most of the information about the decay and subsequent harmonics add progressively less information. Here, we use the first and the second harmonics for each cell decay (*i.e.*, aggregated decay from all pixels inside the cell mask) as alternative features to lifetime fit variables for classification. We also plot the first harmonic phasor plot for visual representation of the cell distributions. The phasor coordinates are calculated as follows.

$$G\left(\omega \right)=\frac{\sum_{t=1}^{Nbins}I\left(t\right)\cdot \cos\left(\omega t\right)}{\sum_{t=1}^{Nbins}I\left(t\right)}$$ (3)

$$S\left(\omega \right)=\frac{\sum_{t=1}^{Nbins}I\left(t\right)\cdot \sin\left(\omega t\right)}{\sum_{t=1}^{Nbins}I\left(t\right)}$$ (4)

where ω=2πnf is the phasor frequency with *f* being the laser repetition frequency (80 MHz) and *n* being the harmonic number (1 or 2). A scale and rotation transformation in the polar coordinates was performed to account for the effect of the instrument response as described in the literature (Ranjit et al., 2018; Martelo et al., 2015).

### Measurement of cytokines and glucose/lactate levels in primary cell media

To validate the activation of lymphocytes in each condition, cytokine levels were measured in the spent media samples collected from control and stimulated conditions (24 h for NK cells, and 72 h for B cells). IFN-γ levels were measured in NK cell media samples using the human IFN-γ DuoSet ELISA kit (R&D Systems). IL-6 levels were measured in B cell media samples using the human IL-6 DuoSet ELISA kit (R&D Systems) (Van Belle et al., 2016; Duddy et al., 2004). The ELISA assay was carried out according to the provided protocol. Plates were incubated overnight with 2 μg/mL IFN-γ or IL-6 capture antibody. The plates were then washed and blocked with a 1% bovine serum albumin solution for 1 h. Following washing, media samples and standards were incubated on the plates for 2 h at room temperature, followed by a 2-h incubation with 200 ng/mL IFN-γ or 50 ng/mL IL-6 detection antibody. Finally, the plates were incubated with streptavidin-conjugated horseradish peroxidase B, then an H_2_O_2_-tetramethylbenzidine substrate solution. The color reaction was stopped at 20 min with a 4 M H_2_SO_4_ solution, and the plates were transferred to a plate reader, where they were read at 450 nm with wavelength correction at 570 nm. Standard curves were calculated from a serial dilution of the standards using a sigmoidal four parameter logistic model. The coefficient of determination R^2^ of the standard curves for the IL-6 and IFN-γ ELISA experiments were 0.9993 and 0.9997, respectively.

To validate that the cells were upregulating aerobic glycolysis in the activated cell populations, commercial kits were used to measure glucose and lactate levels in spent media samples from control and stimulated conditions (24 h for NK cells, and 72 h for B cells). A sample of the media used for the B cells and NK cells described in Table 1 was also evaluated as a control. Glucose and lactate assays were carried out according to the respective protocols for the Glucose Colorimetric/Fluorometric Assay Kit (BioVision) or the Lactate Colorimetric/Fluorometric Assay Kit (BioVision). 0.5 μL of each sample was added to a 96-well plate were an additional 49.5 μL of assay buffer was added, yielding a 100x dilution of the original samples. 50 μL of reaction mix (2 μL probe, 2 μL enzyme mix, and 46 μL assay buffer) was then added to each well to yield a total volume of 100 μL per well. The 96-well trays were left to incubate for 30 min in a dark box at room temperature (glucose assay) or 37°C (lactate assay). The plates were then transferred to a plate reader where glucose or lactate levels were quantified by absorbance at OD 570. Standard curves were calculated from a serial dilution of the standards using an ordinary least squares regression model. The R^2^ of the standard curves for the glucose and lactate assays were 0.9973 and 0.9979, respectively.

### Heatmap, UMAP, and classification

Z-score heatmaps were constructed in R using the Complex Heatmap package (Complex heatmaps reveal patterns and). Clustering of groups or single cells was performed based on the OMI variables and calculated using Ward’s method. Labels for activation and donor were added afterwards and were not included in cluster analysis.

Uniform Manifold Approximation and Projection (UMAP) is a non-linear dimension reduction technique that can be used to visualize high-dimensional data. UMAP projections were made in Python using scikit-learn, UMAP, and Holoviews (McInnes et al., 2018; holoviz/holoviews; Scikit-learn). Unless otherwise noted, each UMAP is a two-dimensional visualization of nine variables (normalized optical redox ratio; NAD(P)H τ_m_, τ_1_, τ_2_, α_1_; FAD τ_m_, τ_1_, τ_2_, α_1_). The UMAP projection was computed using Euclidean distance. The nearest neighbor parameter was set to 15 and the minimum distance was set to 0.4 unless otherwise noted.

Random forest classifiers were trained in Python using scikit-learn to classify activation using the NK cell OMI variables or the B cell OMI variables in their respective cases. The classifiers were trained on a random selection of 70% of the single-cell data and tested on the remaining 30% for B cell or NK cell classifiers (*i.e.*, Figure 2; Figure 4). The phasor classifier was trained on a random selection of 50% of the input data and tested on the remaining 50%. Multiple metrics were used to evaluate the robustness of the classifier, including the receiver operating characteristic (ROC) curve, accuracy, precision, and recall. Classifiers were trained and tested on different random sets of the data to check for consistency in these metrics. Equal cost was given to a misclassified cell regardless of category (*i.e.*, misclassification was not weighted by sample size).

Phasor-based classification was performed using the NAD(P)H and FAD phasor coordinates (G,S) at the laser repetition frequency (80 MHz) and its second harmonic (160 MHz) as features. The phasor coordinates were averaged pixel-wise over each cell mask using pixel intensities as weights to calculate cell-level phasor coordinates. Logistic regression classifiers with a logit link function and random forest classifiers with 100 decision trees were used, and the classifiers were trained on a random selection of 50% of the input data and tested on the remaining 50%. Again, equal cost was given to a misclassified cell regardless of category (*i.e.*, misclassification was not weighted by sample size). Both the phasor and the fit analysis pipelines use the same raw FLIM data and cell masks to calculate cell-level phasor coordinates and fit parameters, respectively. However, the exclusion criteria for the two pipelines are not the same, which results in different final number of cells included in the phasor-based and fit-based classifiers. For example, the phasor pipeline removes low-count (with fewer than 5,000 photons) or small (with fewer than 50 pixels) cells, while the fit analysis also removes cells based on the goodness of the bi-exponential fit (χ^2^ > 1.3).

### Statistical analysis

Statistical analysis was performed using the statannotations package v0.5.0 in Python (Charlier et al., 2022). Differences between groups were tested using Kruskal–Wallis with *post hoc* comparisons test for multiple group comparisons, or a two-tailed unpaired T-test for comparisons of pairs of data. Effect sizes between quiescent and activated cell groups were calculated with Glass’s Delta because comparisons of very large sample sizes of individual cells always pass traditional significance tests unless the population effect size is truly zero (Kaplan et al., 2014). Glass’s Delta is defined as Δ = (µ_control_ - µ_test_)/σ_control_ where values ranging from 0 to 0.2 represent no change, 0.2–0.5 small change, 0.5–0.8 represent moderate change, and anything higher than 0.8 signifies a large change (Sawilowsky, 2009; Glass, 1976).

## Data Availability

The datasets presented in this study can be found in online repositories. The names of the repository/repositories and accession number(s) can be found below: https://github.com/skalalab/schmitz_r-lymphocyte_activation.
