# Supplementary material for: Autofluorescence lifetime imaging classifies human B and NK cell activation state
Source: Front Bioeng Biotechnol. 2025 Apr 4;13:1557021. doi: 10.3389/fbioe.2025.1557021 (PMC12006760; doi:10.3389/fbioe.2025.1557021)
Supplement: Supplementary file 1 [file Presentation1.pdf]

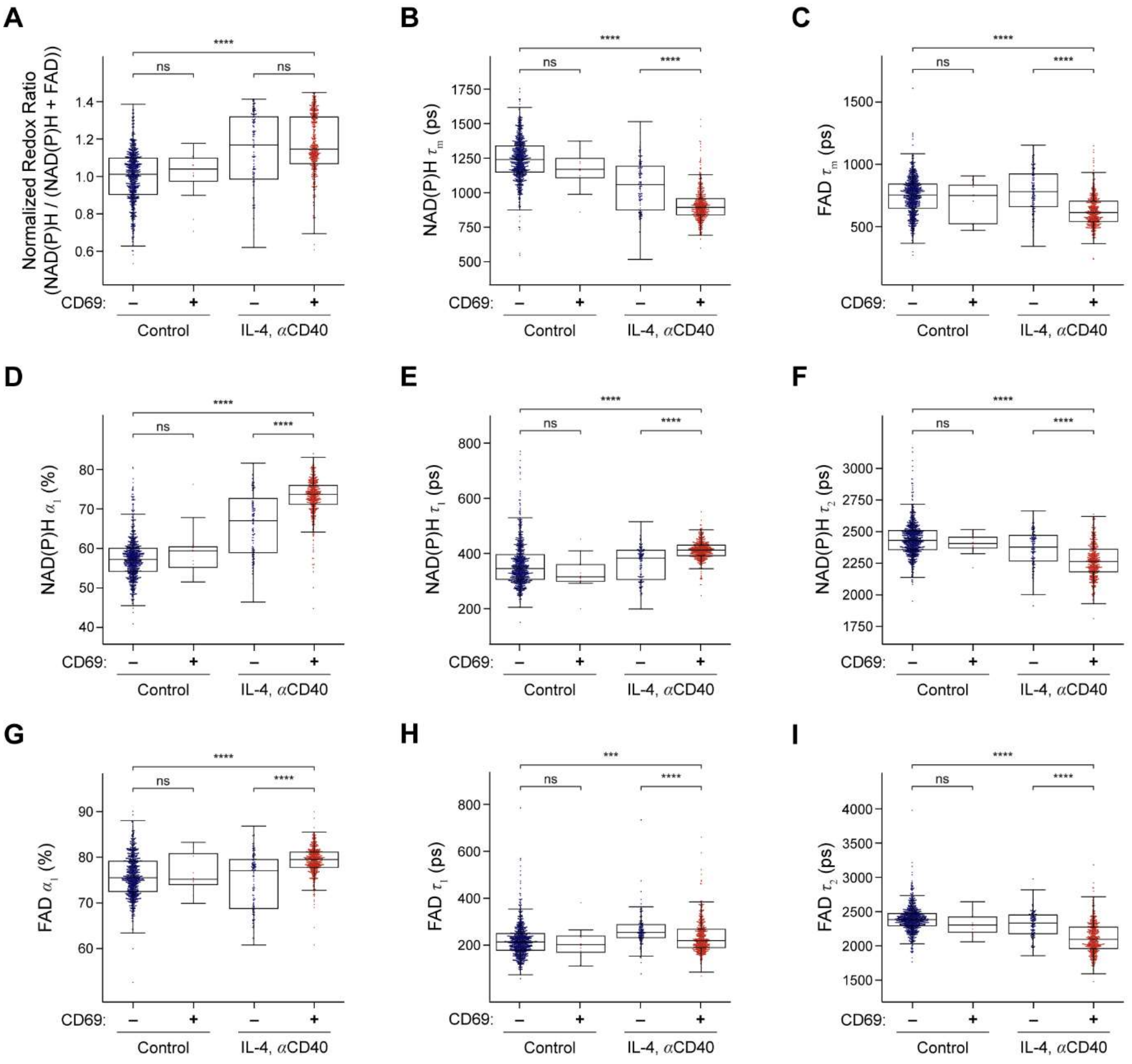

Supplemental Figure 1. OMI of CD69+ and CD69- B cells in control and IL-4 + anti-CD40 stimulated culture. Both CD69+ and CD69- B cells from three donors (A, B, C) in control or stimulated media for each OMI variable: (A) quiescent-normalized optical redox ratio, (B) NAD(P)H mean lifetime, (C) FAD mean lifetime, (D) free NAD(P)H fraction, (E) free NAD(P)H lifetime, (F) protein-bound NAD(P)H lifetime, (G) protein-bound FAD fraction, (H) protein-bound FAD lifetime, (I) free FAD lifetime. Plots display single cell values (dots) overlaid on box-and whisker plots displaying the median, interquartile range (IQR), with whiskers at 1.5*IQR. n = 1352 (749 control CD69- cells, 12 control CD69+ cells, 130 stimulated CD69- cells, 461 stimulated CD69+ cells). ***P<0.001, ****P<0.0001, Kruskal-Wallis with post-hoc comparisons. ns = not significant.

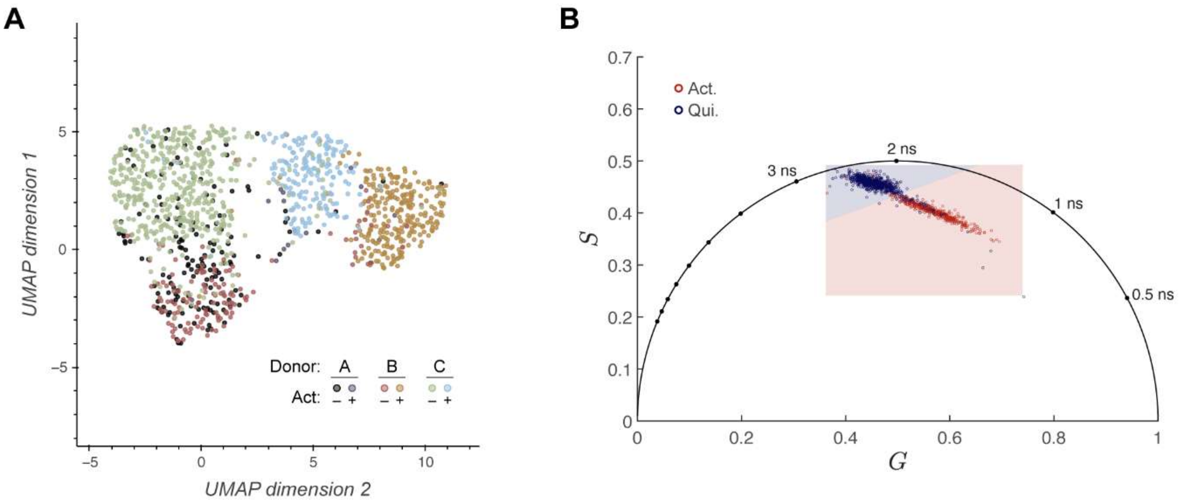

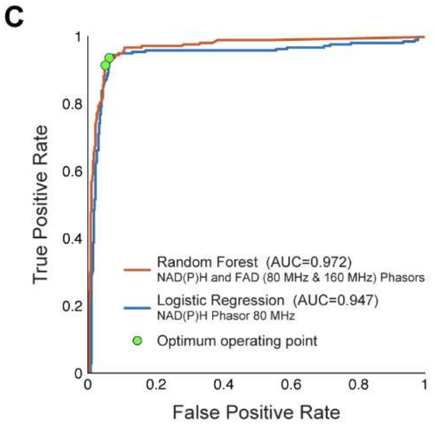

D

		Predicted	
		Act.	Qui.
Actual	Act.	206	19
	Qui.	21	415

Supplemental Figure 2. UMAP of B cells by donor and activation status, and phasor classification of activated B cells. (A) UMAP of B-cells color-coded by donor (A, B, C) and activation status (activated, control). (B) NAD(P)H phasor plot of B cells (red points = activated B cells, blue points = quiescent B cells). Shaded areas show decision boundaries for logistic regression classification of B cell activation based on NAD(P)H phasor. (C-D) ROC curves and confusion matrix for random forest classification of B cell activation based on G and S phasor components for both NAD(P)H and FAD at two frequencies (80MHz, 160MHz) each (8 total variables) or NAD(P)H phasor alone at one frequency (80MHz, 2 total variables). These NAD(P)H and FAD phasors predicted B cell activation with a classification accuracy of 0.939, n = 1323 B cells (451 activated B cells, 872 quiescent B cells) with a 50/50 split for training and test sets.

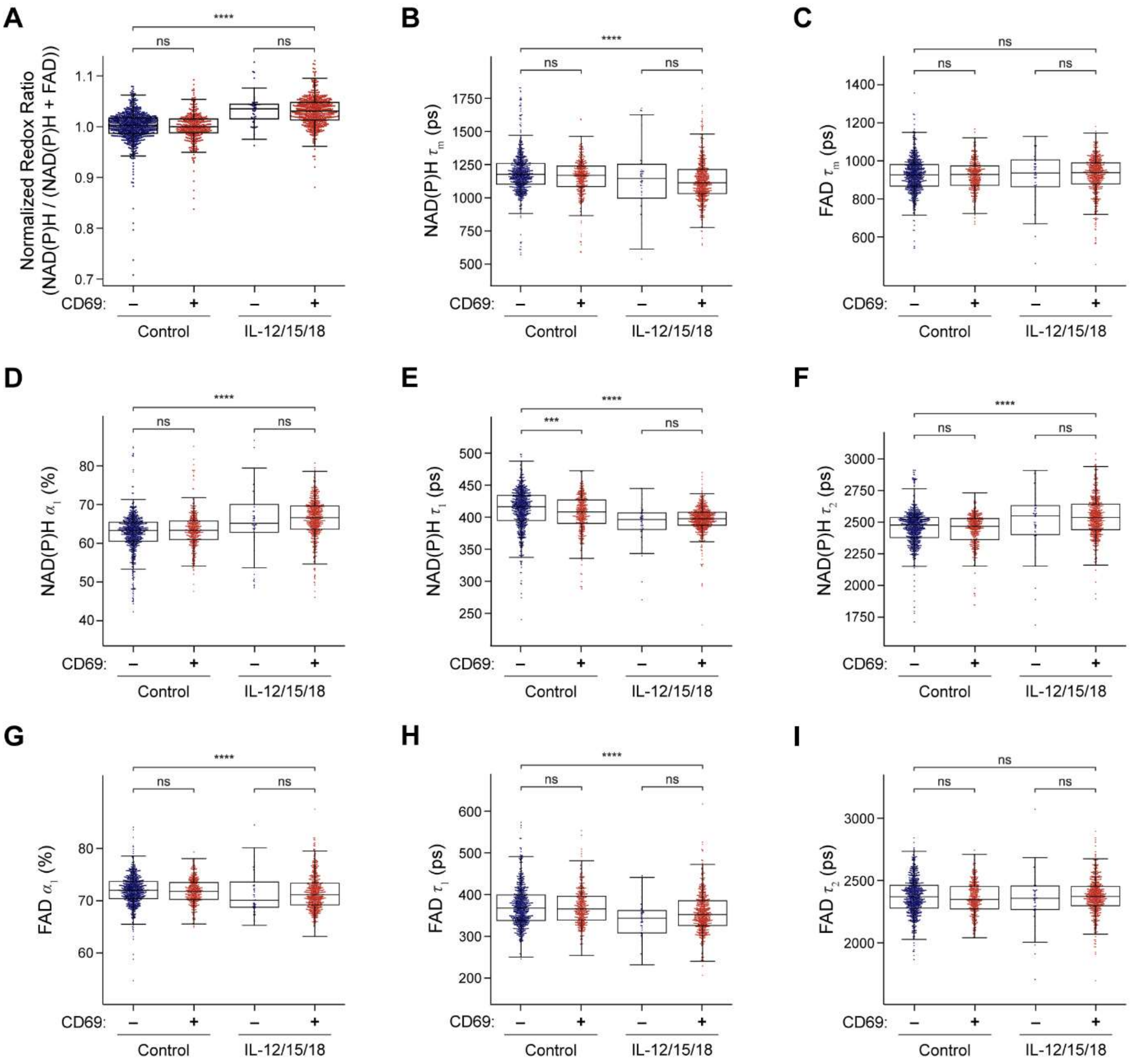

Supplemental Figure 3. OMI of CD69+ and CD69- NK cells in control and IL-12/15/18 stimulated culture. Both CD69+ and CD69- NK cells from three donors (D, E, F) in control or stimulated media for each OMI variable: (A) quiescent-normalized optical redox ratio, (B) NAD(P)H mean lifetime, (C) FAD mean lifetime, (D) free NAD(P)H fraction, (E) free NAD(P)H lifetime, (F) protein-bound NAD(P)H lifetime, (G) protein-bound FAD fraction, (H) protein-bound FAD lifetime, (I) free FAD lifetime. Each point is a single cell and overlaid are box-and whisker plots displaying the median, interquartile range (IQR), with whiskers at 1.5*IQR. n = 1642 cells (667 control CD69- cells, 372 control CD69+ cells, 49 stimulated CD69- cells, 554 stimulated CD69+ cells). ****P<0.0001, Kruskal-Wallis with post-hoc comparisons. ns = not significant.

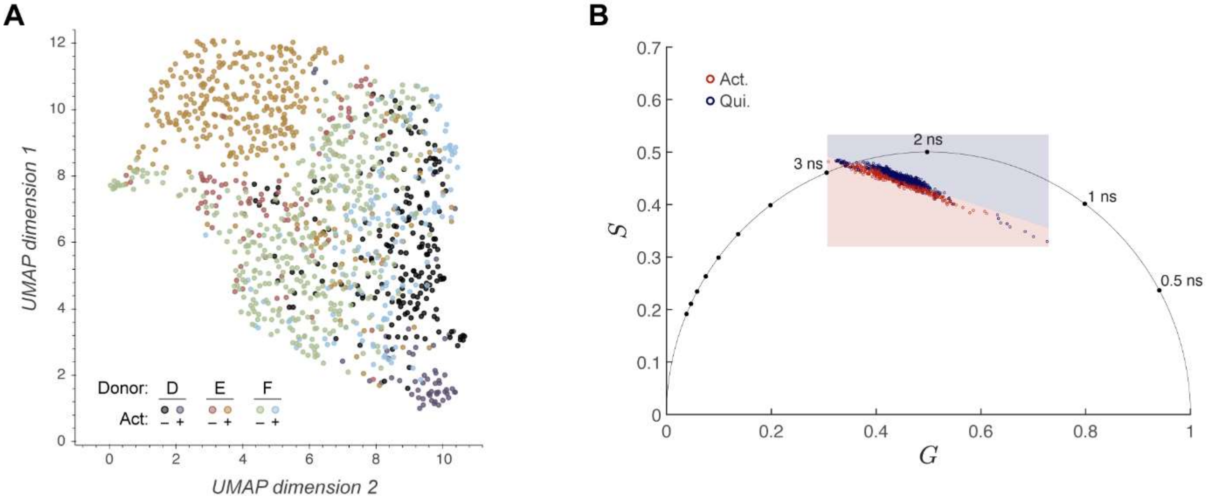

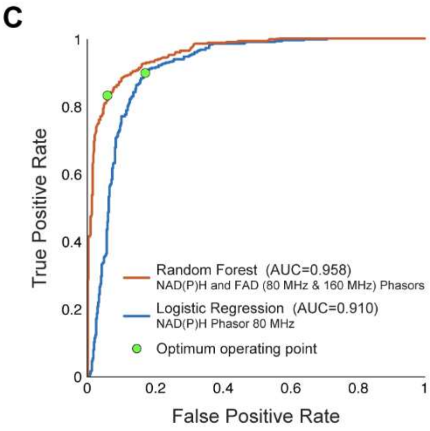

D

		Predicted	
		Act.	Qui.
Actual	Act.	324	66
	Qui.	28	452

Supplemental Figure 4. UMAP of NK cells by donor and activation status, and phasor classification of activated NK cells. (A) UMAP of NK cells color-coded by donor (D, E, F) and activation status (activated, quiescent). (B) NAD(P)H phasor plot of NK cells from Fig. 3F-I (red = activated NK cells, blue = quiescent NK cells). Shaded areas show decision boundaries for logistic regression classification of NK cell activation based on NAD(P)H phasor. (D) ROC curves and confusion matrix for random forest classification of NK cell activation based on G and S phasor components for both NAD(P)H and FAD at two frequencies (80MHz, 160MHz) each (8 total variables) or NAD(P)H phasor alone at one frequency (80MHz, 2 total variables). These NAD(P)H and FAD phasors predicted NK cell activation with a classification accuracy of 0.892, n = 1742 cells (781 activated NK cells, 961 quiescent NK cells) with a 50/50 split for training and test sets.

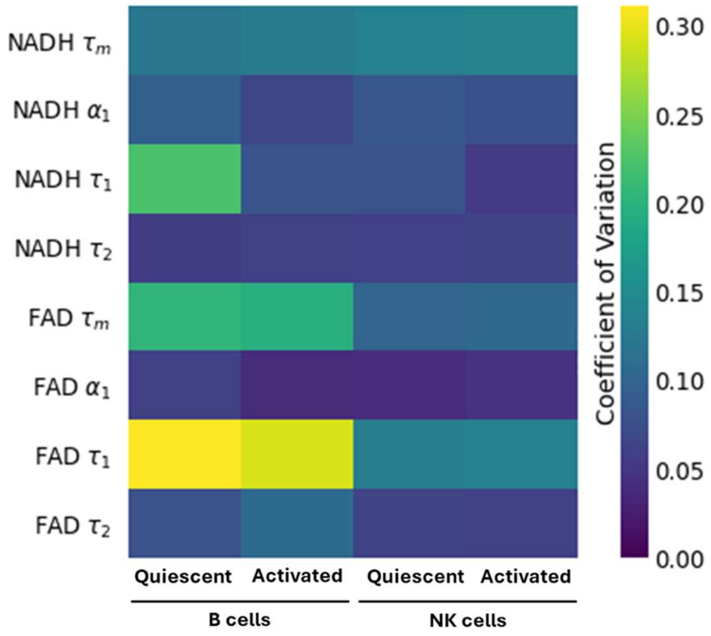

Supplemental figure 5. Single cell heterogeneity in fluorescence lifetime variables for B and NK cells. Heatmap of the coefficient of variation (COV = standard deviation / mean) of NAD(P)H and FAD fluorescence lifetime variables for activated and quiescent B and NK cells. B cells are from 3 donors (A, B, C), n = 1210 cells (461 activated B cells, 749 quiescent B cells). NK cells are from 3 donors (D, E, F), n = 1221 cells (554 activated NK cells, 667 quiescent NK cells).
